# Circular RNA circCHSY1 silencing inhibits the malignant progression of esophageal squamous cell carcinoma

**DOI:** 10.1007/s12672-024-00935-z

**Published:** 2024-03-21

**Authors:** Haiquan He, Ying Chen, Hanping Liang, Weibi Che, Huilong Chen, Ying Chen, Fengyuan Peng, Bomeng Wu

**Affiliations:** Department of Thoracic Surgery, Gaozhou People’s Hospital, No. 89, Xiguan road, Gaozhou, 525200 China

**Keywords:** ESCC, circCHSY1, miR-1229-3p, TCTN1

## Abstract

**Background:**

CircRNAs play a crucial role in the regulation of various cancers. This study aims to investigate the involvement of circCHSY1 in the development of esophageal squamous cell carcinoma (ESCC).

**Methods:**

RNA levels were quantified using qRT-PCR, and protein levels were measured by western blot. The stability of circCHSY1 was analyzed using RNase R. The functional effect of circCHSY1 on cell behavior was evaluated by CCK-8, EdU, flow cytometry, transwell, tube formation, and xenograft tumor model assays. The associations among circCHSY1, miR-1229-3p, and Tectonic-1 (TCTN1) were certified by bioinformatics analysis, dual-luciferase reporter assay, and RNA pull-down assay.

**Results:**

CircCHSY1 was up-regulated in both ESCC tissues and cell lines in comparison with the control groups. Knockdown of circCHSY1 inhibited the proliferation, migration, invasion, and tube formation and promoted apoptosis of ESCC cells. Mechanistically, circCHSY1 targeted miR-1229-3p, which was downregulated in ESCC tissues and cells. Inhibition of miR-1229-3p attenuated the effects mediated by circCHSY1 suppression. Besides, miR-1229-3p bound to TCTN1, and TCTN1 overexpression restored miR-1229-3p-induced effects in ESCC cells. Animal experiments revealed that circCHSY1 silencing suppressed tumor tumorigenesis in vivo.

**Conclusion:**

CircCHSY1 contributed to ESCC cell malignancy, and the underlying mechanism involved the circCHSY1/miR-1229-3p/TCTN1 axis, providing potential therapeutic targets for ESCC.

## Introduction

More than 450,000 new cases are diagnosed with esophageal cancer every year, placing it ninth among all cancer types [1, 2]. Pathologically, esophageal squamous cell carcinoma (ESCC) accounts for over 90% of these cancers [3]. Although current treatment methods, such as drug therapy and targeted therapy, can significantly improve the therapeutic effect for ESCC patients, the prognosis remains poor, especially in the advanced stage [4, 5]. Therefore, it is necessary to investigate the molecular pathogenesis of ESCC and identify molecular markers and therapeutic targets for the disease.

CircRNAs were first discovered in 1976 [6]. In 1991, Nigro et al. identified their presence in human cells when studying the tumor suppressor gene DCC [7]. The covalent closed-loop structure of circRNAs makes them more stable than the linear RNAs within cells [8]. Importantly, circRNAs are expressed in specific cell types or pathological conditions [9]. Some circRNAs serve as prognostic biomarkers for various cancers, including glioma, hepatocellular carcinoma, and ESCC [10–12]. The regulatory role of circRNA in ESCC requires further exploration. In a previous report, Wang et al. reported the dysregulation of circCHSY1 (circRNA ID hsa_circ_0005019) in ESCC through qRT-PCR analysis [13]. However, research on circCHSY1 is still relatively rare.

MicroRNAs (miRNAs) are non-coding small molecules that play a crucial role in tumorigenesis. Abnormal expression of certain miRNAs has been detected in ESCC tissues, contributing to the development and progression of the disease. For instance, miR-375 is highly expressed in ESCC, promoting tumor cell growth and metastasis [14]. Moreover, miR-34a inhibited ESCC cell proliferation and migration [15]. Another miRNA, miR-1229-3p, was found to promote the progression of some cancers such as gastric cancer [16] and breast cancer [17]. However, the role of miR-1229-3p in ESCC progression remains unclear.

Herein, we analyzed circCHSY1 and miR-1229-3p expression, and determined the role of circCHSY1 in ESCC cell malignancy. Additionally, the present work investigated whether the regulation of circCHSY1 in ESCC progression involved miR-1229-3p.

## Materials and methods

### Human samples

The study was carried out with the approval of the Ethics Committee of Gaozhou People’s Hospital and was performed in accordance with the ethical standards as laid down in the 1964 Declaration of Helsinki 56 pairs of fresh ESCC tissues and adjacent normal tissues were obtained from this hospital. Informed consent was obtained from all participants before sample collection. The collected tissue specimens were stored at − 80 °C.

### Cell culture and transfection

Normal esophageal epithelial cells (HET-1A) were acquired from ATCC (Manassas, VA, USA). TE-1, Eca-109, KYSE150, and HUVEC cell lines were obtained from Procell (Wuhan, China), while KYSE70 cells were provided by TongPai Biotechnology (Shanghai, China). KYSE70 cells and KYSE150 cells were maintained in RPMI-1640 medium, and other cells were cultivated in a DMEM medium. During cell culture, medium was supplemented with 1% penicillin/streptomycin (Procell) and 10% fetal bovine serum (FBS). The culture conditions were 37 °C, 5% CO_2_, and 95% humidity.

SiRNAs for circCHSY1 (si-circCHSY1-1 and si-circCHSY1-2), shRNA against circCHSY1 (sh-circCHSY1), miR-1229-3p mimic and inhibitor, Tectonic-1 (TCTN1) overexpressed plasmid (pcDNA-TCTN1) and their negative controls were all provided by GenePharma (Shanghai, China). When the cell aggregation rate reached 80%, 50 nM of miRNA mimic, different substances (20 pmoL of siRNA, 0.8 µg of plasmids, and 100 nM of miRNA inhibitor) were used for cell transfection by Lipofectamine^™^ 3000 (Invitrogen, Carlsbad, CA, USA) depending on the experimental requirements.

### qRT-PCR

In line with the guidebook of GoldenstarTM RT6 cDNA Synthesis Kit (Chinese Indigo), the isolated RNA by TRIzol^™^ was used to synthesize complementary DNA. Premix Ex Taq^™^ II (Chinese Indigo) was used for real-time PCR. RNA quantification was performed by the 2^−∆∆Ct^ method. Primer’s sequences used in this paper are listed in Table 1.

**Table 1:** Primer sequences for qRT-PCR

Name		Primers (5′-3′)
circCHSY1	Forward	TCTTATGAGAACATGGTCCAAGAC
	Reverse	CCACACCCCGTAGTGGC
CHSY1	Forward	CAGGAACTTTCTCTTCGTGGGA
	Reverse	TCCAAGTAGTGGTCGTGCAT
miR-1229-3p	Forward	GCCGAGCTCTCACCACTGCCCTC
	Reverse	AGTGCAGGGTCCGAGGTATT
TCTN1	Forward	ATGAGGTGAGCCTGAACTTGA
	Reverse	CTCTGTCTCAAGGAACCCAGT
GAPDH	Forward	GGTCACCAGGGCTGCTTT
	Reverse	GGAAGATGGTGATGGGATT
U6	Forward	CTTCGGCAGCACATATACT
	Reverse	AAAATATGGAACGCTTCACG

### RNase R digestion

Three μg of RNA was incubated with RNase R (Epicentre Biotechnologies, Madison, WI, USA) for 20 min under normal conditions. Expression of circCHSY1 and its linear gene CHSY1 was measured by qRT-PCR.

### CCK8 test

1 × 10^4^ TE-1 or Eca-109 cells for each assay were respectively planted onto the 96-well plates and transfected with different substances (si-circCHSY1, si-NC, miRNA inhibitors, inhibitor control, miRNA mimics, mimic control, pcDNA-TCTN1 and pcDNA-NC) into the cells. Cell proliferative ability was then analyzed following the suggestion of CCK8 kit (Beyotime, Shanghai, China).

### EdU assay

EdU Imaging Detection Kit (KeyGEN, Nanjing, China) was utilized to assess cell proliferation. ESCC cells (5 × 10^4^/well) with different transfections were seeded in 96-well plates and subsequently went through incubation with an EdU working reagent. Subsequently, the cells were fixed with paraformaldehyde (4%) and infiltrated with Triton X-100 solution (0.5%). After the addition of Click-iT EdU reaction buffer (100 µL), images of EdU-positive cells were recorded under a fluorescence microscope.

### Flow cytometry

Cells with different substances (si-circCHSY1, si-NC, miRNA inhibitors, inhibitor control, miRNA mimics, mimic control, pcDNA-TCTN1, and pcDNA-NC) were cultured at 37 °C for 48 h. 1 × 10^5^ cells were harvested and suspended in 5 μL of Annexin V-FITC and 10 μL of PI (Beyotime) for 10 min. Apoptotic cells were detected by flow cytometry (Countstar, Shanghai, China).

### Transwell assay

Matrigel (Millipore, Billerica, MA, USA) was utilized for cell invasion analysis. In simple terms, 1 × 10^5^ of ESCC cells with different substances (si-circCHSY1, si-NC, miRNA inhibitors, inhibitor control, miRNA mimics, mimic control, pcDNA-TCTN1, and pcDNA-NC) were added to the upper chambers. After 24 h of culture, the invaded cells were finally photographed under a microscope.

### Tube formation assay

HUVEC cells (3 × 10^4^ cells/well) were treated with si-circCHSY1, si-NC, anti-miR-1229-3p, miR-NC, pcDNA-TCTN1, pcDNA-NC, anti-miR-NC or miR-1229-3p, alone or jointly. The cells were suspended into 96-well plates coated with 60 μL Matrigel (Millipore) and cultured with supernatant of ESCC cells for 5 h. Then, a microscope was used to determine the number of capillary-like branches.

### Western blot

Proteins were extracted from cells and tissues using RIPA lysis solution (Beyotime). The proteins were separated by 5% SDS-PAGE and then transferred onto membranes. The membranes were blocked with non-fat milk for 1.5 h and incubated overnight with primary antibodies including anti-PCNA (ab92552, 1:1000, Abcam, Cambridge, MA, USA), anti-Bax (ab32503, 1:1000, Abcam), anti-Bcl-2 (ab182858, 1:1000, Abcam), anti-TCTN1 (15,004-1-AP, 1:500, proteintech, Chicago, Illinois, USA) and anti-GAPDH (ab9485, 1:1000, Abcam). The protein bands were visualized using ECL substrates (Millipore).

### Dual-luciferase reporter gene assay

Wild (WT) or mutant (MUT) circCHSY1 (or TCTN1) containing the potential binding sites for miR-1229-3p were inserted into the psi-CHECK vector (Promega, Madison, WI, USA). TE-1 and Eca-109 cells were subsequently co-transfected with the constructed plasmids described above with miR-1229-3p or miR-NC. The cells were divided into groups including circCHSY1^WT^ + miR-1229-3p group, circCHSY1^WT^ + miR-NC group, circCHSY1^MUT^ + miR-1229-3p group, circCHSY1^MUT^ + miR-NC group, TCTN1 3’UTR^WT^ + miR-1229-3p, TCTN1 3’UTR^WT^ + miR-NC, TCTN1 3’UTR^MUT^ + miR-1229-3p, and TCTN1 3’UTR^MUT^ + miR-NC group. After 48 h, luciferase activity was analyzed using a dual-luciferase assay system.

### RNA pull-down assay

Eca109 and TE-1 cells were collected and incubated in a RIPA lysis buffer. The lysate was incubated with biotin-labeled oligonucleotide probes and Streptavidin-coupled Dynabeads (Invitrogen). The magnetic beads were incubated at 4 °C for 3 h and the combined RNA in the complex was purified using TRIzol. MiR-1224-3p, miR-1253, and miR-1229-3p expression were assessed by qRT-PCR.

### Nucleus-cytoplasm fractionation

PARIS^™^ kit (Invitrogen) was applied to determine the position of circCHSY1 in cells. The cytoplasmic and nuclear components of ESCC cells were isolated and collected for qRT-PCR analysis of circCHSY1 expression.

### In vivo studies

Specific pathogen-free BALB/c nude female mice (n = 10) aged 4–5 weeks weighing 18–22 g were purchased from Hunan Slyke Jingda Experimental Animal Co., LTD (Changsha, China) and maintained in the absence of specific pathogens. The experiments were approved by the Administrative Panel on Laboratory Animal Care of Gaozhou People’s Hospital. All animal procedures were carried out following the National guidelines of the Animal Care and Use of laboratory animals, the ARRIVE guidelines and the Basel Declaration. The maximal tumor size permitted by the Ethics Committee of Gaozhou People’s Hospital is not exceed 2000 mm^3^ in size and 20 mm in diameter. The generated xenograft tumors in this study were not exceeded this range. Two groups of mice were injected subcutaneously with TE-1 cells stably expressing sh-NC or sh-circCHSY1. The density of cell suspension was adjusted to 1 × 10^6^ cells/mL, and each mouse was injected with the cell suspension (200 μL) to establish an in vivo model. Tumor volume was recorded weekly. After 4 weeks, the mice were anesthetized using pentobarbital sodium, and the tumor was dissected for tumor weight and gene expression analysis. Confounders were not controlled in this experiment. Immunohistochemistry (IHC) was conducted to analyze PCNA, Bax, Bcl-2, and Ki67 expression as instructed [18]. Antibodies were obtained from Abcam (Shanghai) Trading Co., LTD.

### Statistical analyses

Data in this study were analyzed by GraphPad Prism version 5.0 and presented as mean ± standard deviation. Distribution normality was evaluated using the Shapiro-Wilk normality test. The Student’s *t-test* was used to analyze the difference between 2 groups. Analysis of variance was performed to analyze data among three or more groups. *P*-value less than 0.05 meant a significant difference.

## Results

### CircCHSY1 was up-regulated in ESCC tissues and cells

**Fig. 1:**
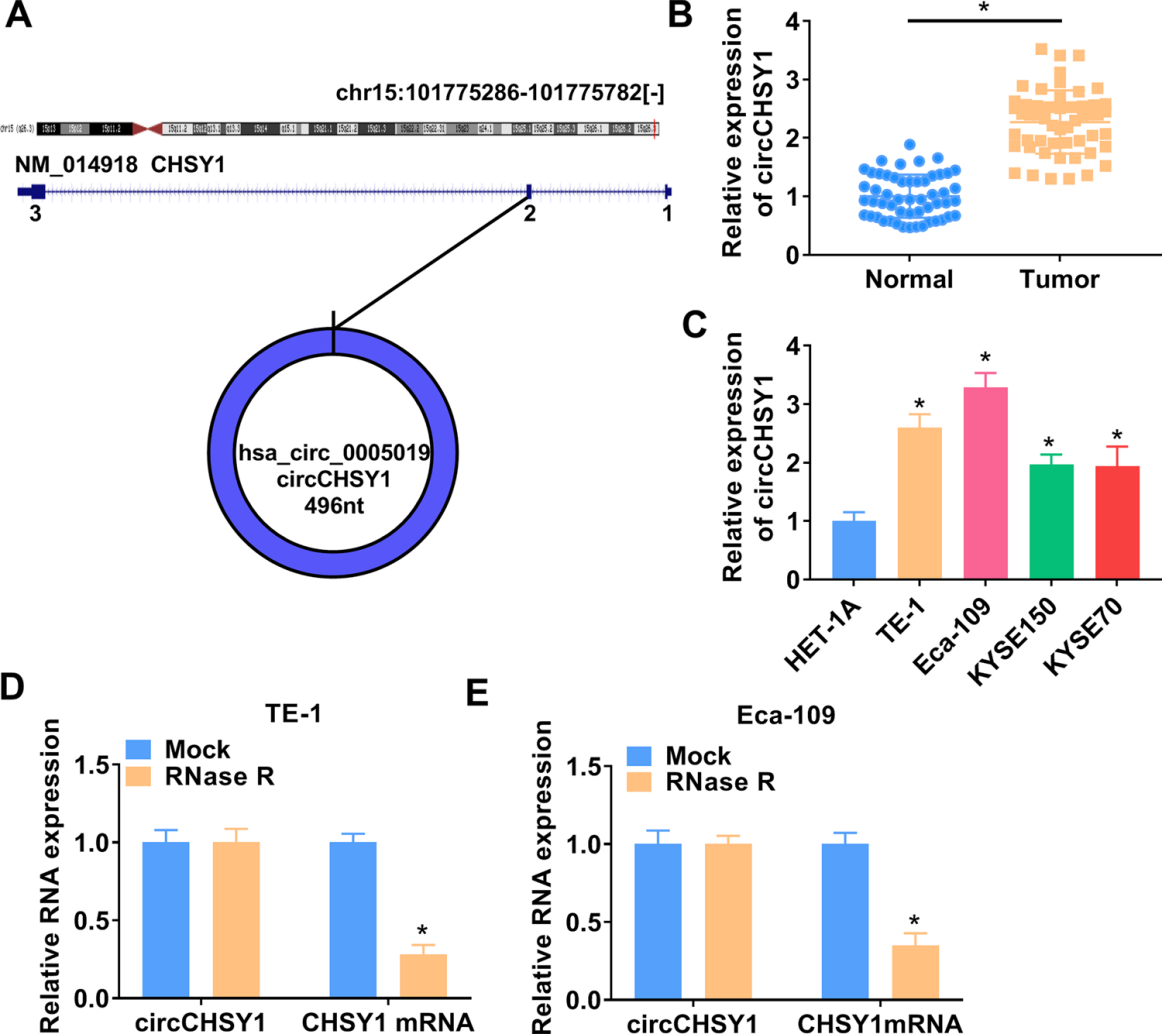
CircCHSY1 was up-regulated in ESCC tissues and cells. **A** The spliced mature sequence of circCHSY1 originated from the CHSY1 gene. **B** The expression of circCHSY1 in normal tissues (N = 53) and ESCC tissues (N = 53) was detected by qRT-PCR. **C** Expression of circCHSY1 in ESCC cell lines (TE-1, Eca-109, KYSE150 and KYSE79) was tested by qRT-PCR. **D**, **E** The expression of circCHSY1 and CHSY1 mRNA in ESCC cells was detected by qRT-PCR. The assay was performed with three independent biological replicates. *P < 0.05

CircCHSY1 was generated through the back-splicing of exon 3, which was located on the CHSY1 gene (Fig. 1A). Compared with normal adjacent tissues, circCHSY1 was significantly increased in tumor tissues (Fig. 1B). Moreover, ESCC cell lines (TE-1, Eca-109, KYSE150 and KYSE79) showed a higher expression of circCHSY1 than HET-1A cells (Fig. 1C). TE-1 and Eca-109 cells were selected for the following study due to higher expression of circCHSY1 in the two types of cells. RNase R treatment assay revealed that circCHSY1 was almost unaffected, but CHSY1 mRNA was reduced (Fig. 1D and E), indicating the circular character of circCHSY1. These data demonstrated that circCHSY1 might be associated with ESCC progression.

### Silencing of circCHSY1 suppressed ESCC cell malignancy

**Fig. 2:**
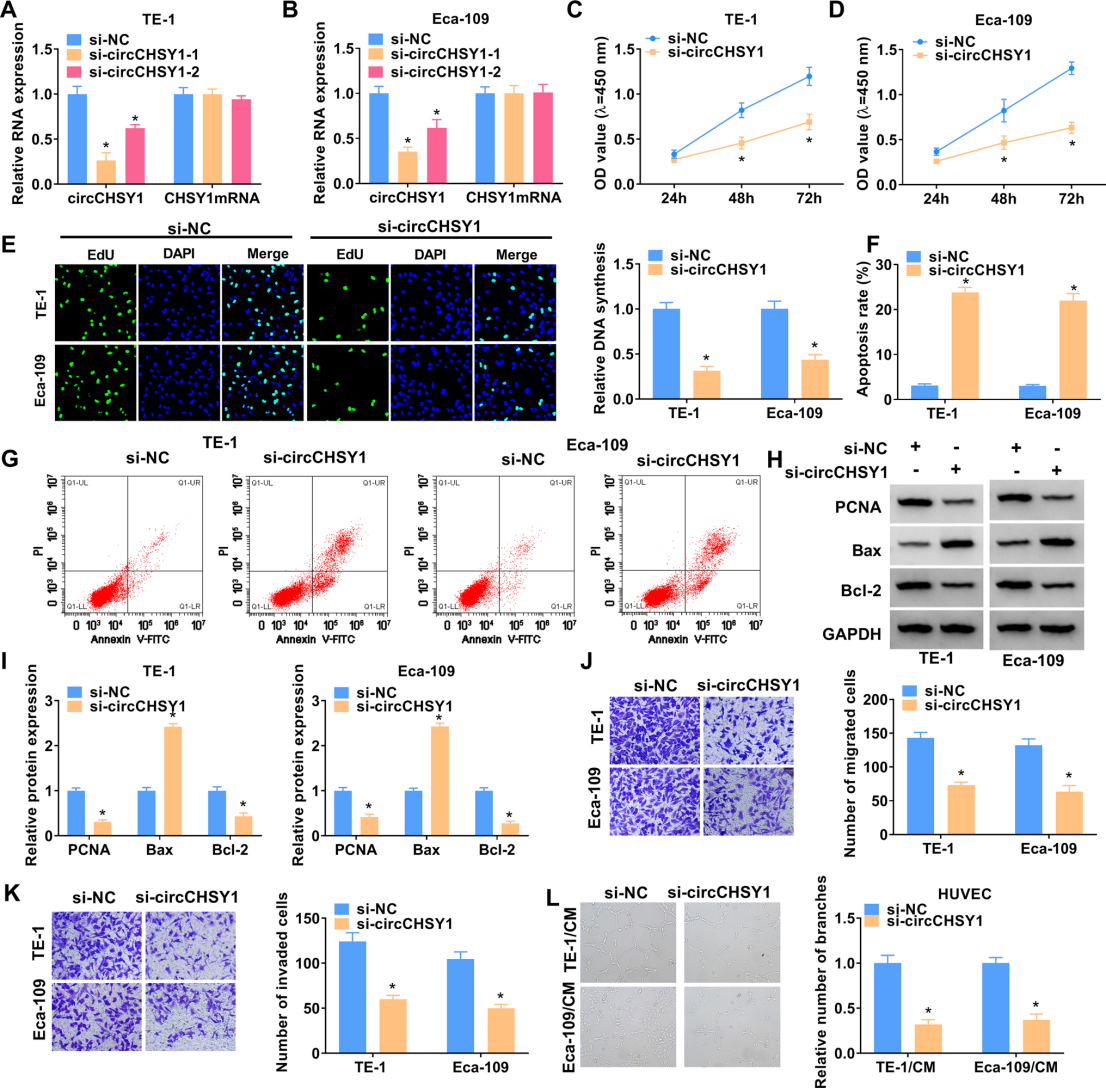
Silencing of circCHSY1 suppressed the malignant biological behaviors of ESCC cells. **A**, **B** Expression of circCHSY1 and CHSY1 were confirmed by qRT-PCR in ESCC cells transfected with si-NC, si-circCHSY1-1 or si-circCHSY1-2. **C**, **D** The viability of TE-1 and Eca-109 cells transfected with si-NC or si-circCHSY1 was assessed by CCK-8 assay. **E** Cell proliferation of TE-1 and Eca-109 cells was detected by the EdU method. **F**, **G** Flow cytometry was administrated to assess cell apoptosis. **H**, **I** The protein levels of PCNA, Bax, and Bcl-2 were examined by western blot. **J**, **K** The migration and invasion ability of the cells were reflected by the transwell assay. **L** The tube formation experiment was used to detect the effect on the angiogenesis of HUVEC cells. The scale bar represents 100 μm. The assay was performed with three independent biological replicates. *P < 0.05

The functional experiments were performed using siRNA of circCHSY1 due to its high expression in ESCC cells. Firstly, the interference efficiency of circCHSY1 siRNAs was validated, and these two siRNAs significantly reduced circCHSY1 expression without affecting linear genes (Fig. 2A and B). si-circCHSY1-1 (named as si-circCHSY1 in the following studies) was chosen for subsequent investigation due to its stronger inhibitory effect on circCHSY1 expression. CircCHSY1 knockdown significantly reduced cell viability, as evidenced by CCK-8 analysis (Fig. 2C and D). Similar results were obtained in the EdU test, which showed a decrease in cell proliferation after circCHSY1 silencing (Fig. 2E). The number of apoptotic cells was increased after transfection with si-circCHSY1 (Fig. 2F and G). When circCHSY1 expression was down-regulated, Bax was increased significantly, while PCNA and Bcl-2 levels were decreased significantly (Fig. 2H and I). In addition, circCHSY1 silence could inhibit cell migration, invasion, and tube formation (Fig. 2J–L). These findings suggest that inhibition of circCHSY1 has anti-tumor effects in ESCC cells.

### CircCHSY1 bound to miR-1229-3p

**Fig. 3:**
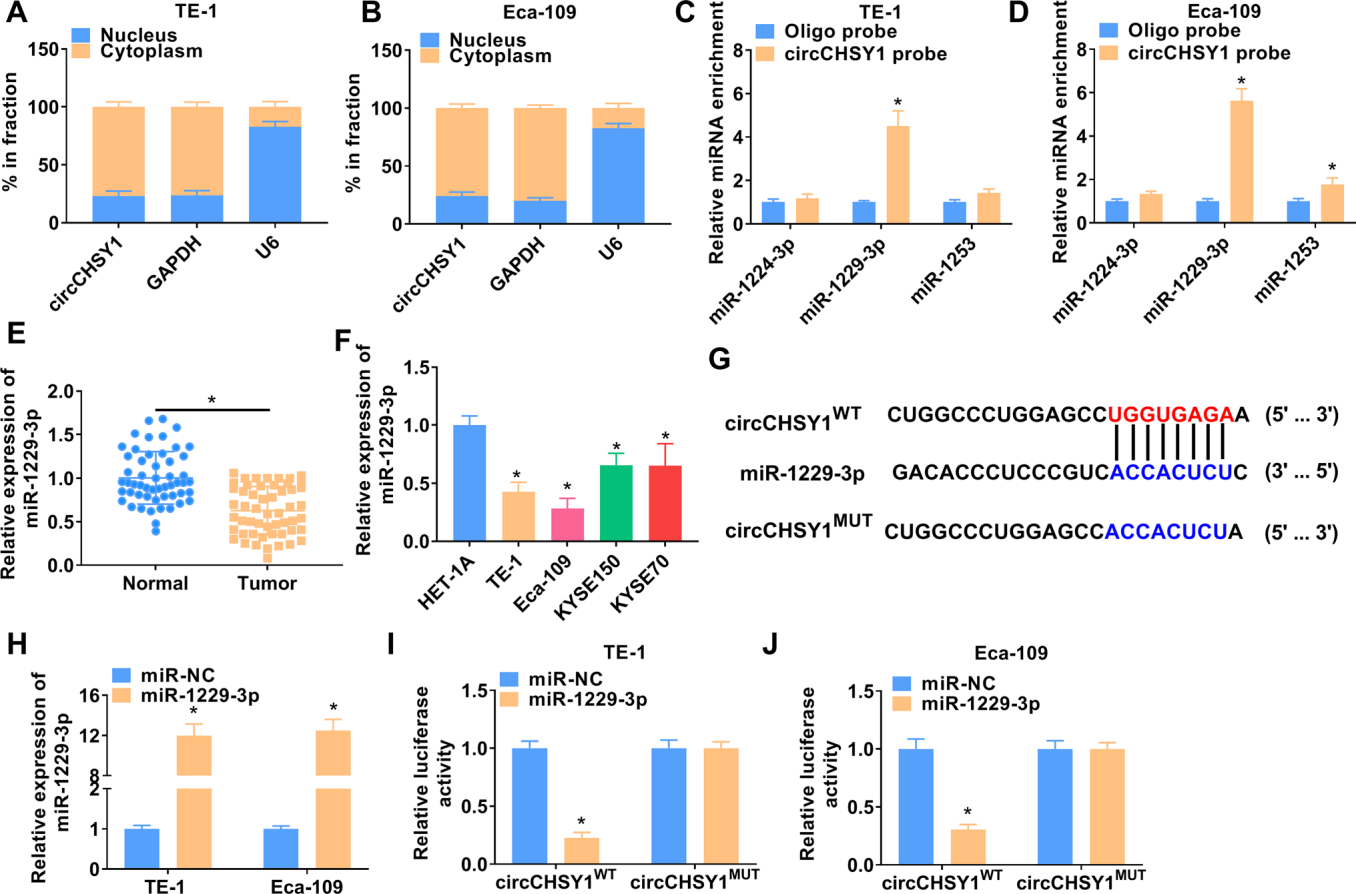
CircCHSY1 served as a sponge for miR-1229-3p. **A**, **B** The expression levels of GAPDH, U6 and circCHSY1 were determined by qRT-PCR in the cytoplasmic and nuclear parts of ESCC cells. **C**, **D** RNA pull-down assay was applied to identify the association of circCHSY1 with miR-1224-3p, miR-1253, and miR-1229-3p. **E** MiR-1229-3p expression in normal tissues (N = 53) and ESCC tissues (N = 53) was analyzed by qRT-PCR. **F** The level of miR-1229-3p in cells was detected with qRT-PCR. **G** The putative complementary sites of circCHSY1 and miR-1229-3p were predicted by bioinformatics analysis. **H** qRT-PCR was performed to analyze miR-1229-3p expression in ESCC cells transfected with miR-NC and miR-1229-3p. **I**, **J** Dual-luciferase reporter assays were implemented to analyze the interaction between circCHSY1 and miR-1229-3p. The assay was performed with three independent biological replicates. *P < 0.05

CircCHSY1 was mainly presented in the cytoplasm (Fig. 3A and B). Through the prediction of the circinteractome online database, we found that circCHSY1 is potentially bound to miR-1224-3p, miR-1253, and miR-1229-3p. As shown in Fig. 3C and D, only miR-1229-3p expression was significantly upregulated in the circCHSY1 probe group among the three miRNAs. Thus, miR-1229-3p was chosen for the present study. MiR-1229-3p was significantly reduced in ESCC tissues and cells (Fig. 3E and F), so we speculated whether circCHSY1 could target to miR-1229-3p. The binding sites of circCHSY1 for miR-1229-3p are shown in Fig. 3G. The success of miR-1229-3p overexpression is presented in Fig. 3H. As expected, miR-1229-3p overexpression decreased the luciferase activity of circCHSY1^WT^, which further proved the combination of the two RNAs (Fig. 3I and J).

### MiR-1229-3p inhibitor restored tumorigenesis in circCHSY1-deficient cells

**Fig. 4:**
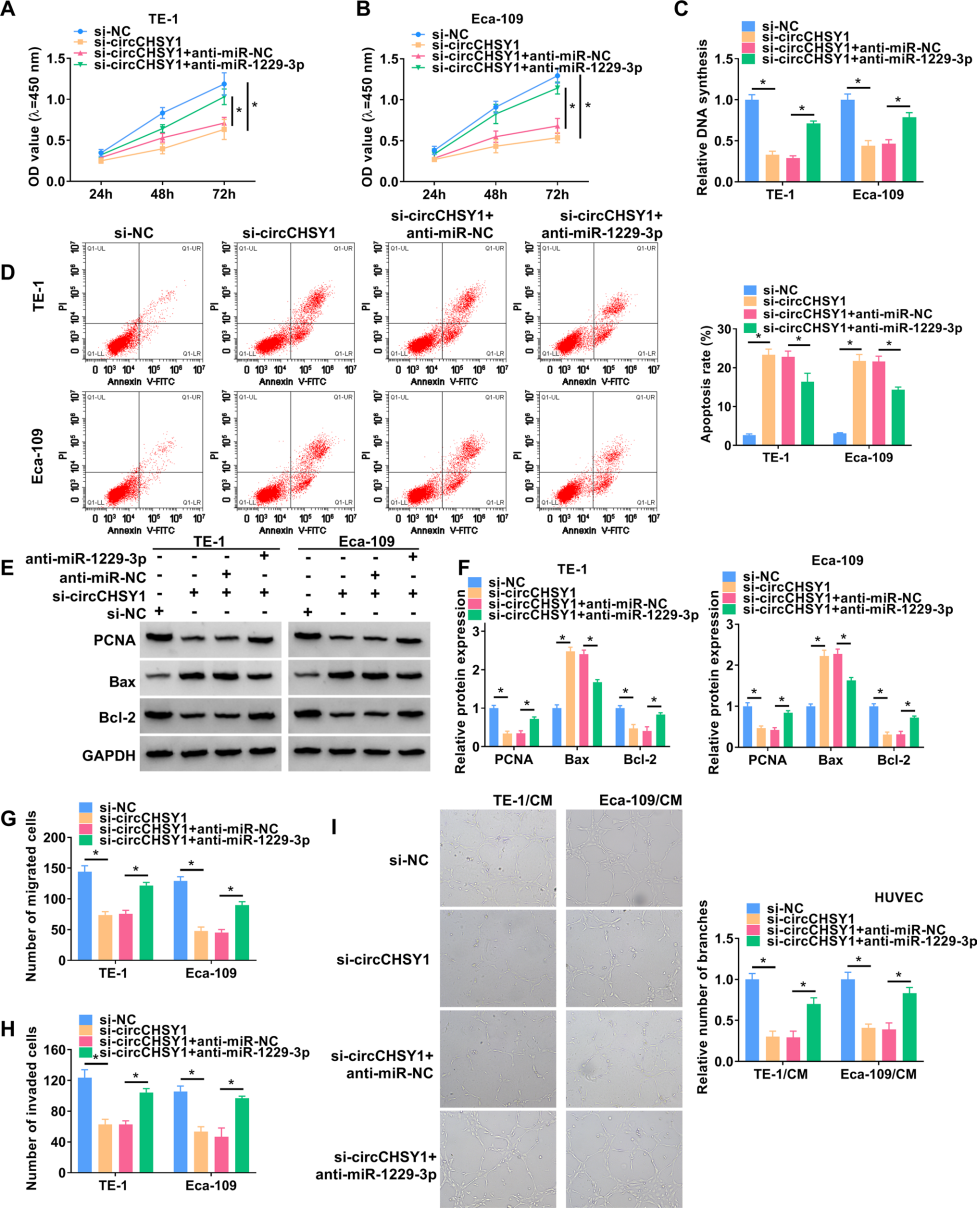
MiR-1229-3p inhibitor restored tumorigenesis in circCHSY1-deficiency cells. **A**–**C** CCK-8 assay **A**, **B** and EdU assay **C** were performed to detect the proliferation of TE-1 and Eca-109 cells. **D** Flow cytometry was applied to assess the apoptosis rate of TE-1 and Eca-109 cells. **E** and **F** The protein expression of PCNA, Bax and Bcl-2 was examined by western blot. **G**–**I** The ability of cell migration, invasion and angiogenesis was detected by transwell assay and tube formation assay. The assay was performed with three independent biological replicates. *P < 0.05

The data of CCK-8 (Fig. 4A and B) and EdU analysis (Fig. 4C) showed that circCHSY1 absence inhibited cell proliferation, but transfection of miR-1229-3p inhibitor reversed this inhibition. As shown in Fig. 4D, the cell apoptosis rate was significantly enhanced by decreasing the expression of circCHSY1, while anti-miR-1229-3p reduced the effect. At the protein level, the circCHSY1 deficiency increased Bax protein expression and decreased PCNA and Bcl-2 protein levels, while anti-miR-1229-3p attenuated these effects (Fig. 4E and F). In addition, knockdown circCHSY1 inhibited cell migration, invasion, and angiogenesis. However, these effects were mitigated by co-transfection with miR-1229-3p inhibitors (Fig. 4G–I).

### MiR-1229-3p directly targeted TCTN1

**Fig. 5:**
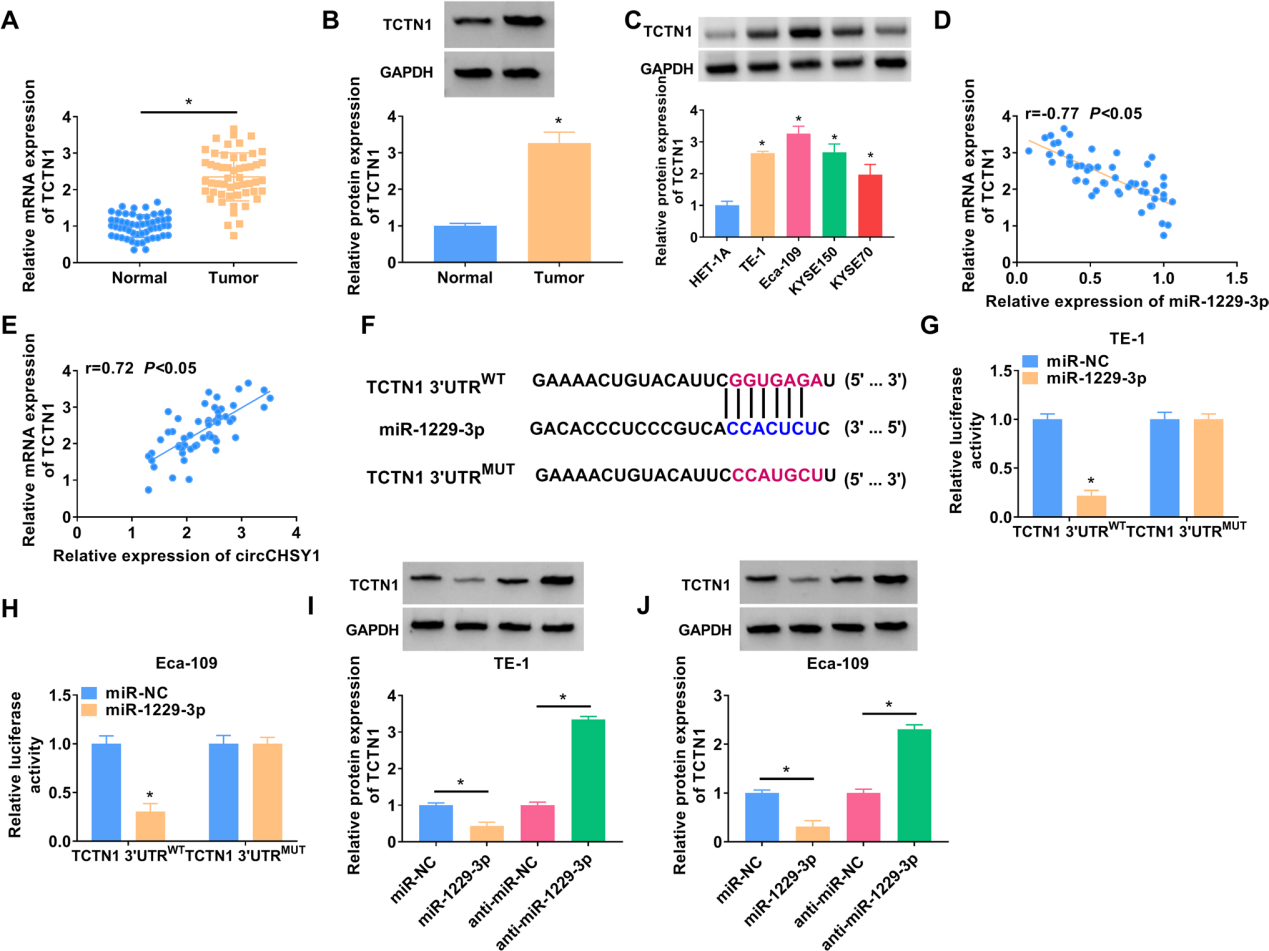
MiR-1229-3p directly targeted TCTN1. **A**, **B** The mRNA and protein expression of TCTN1 in tissues was measured by qRT-PCR and western blot. **C** The expression of TCTN1 in ESCC cells and HET-1A cells was detected by western blot. **D**, **E** The linear associations between TCTN1 and miR-1229-3p and circCHSY1 were analyzed by Spearman’s correlation coefficient. **F** The binding sites of miR-1229-3p and TCTN1 were predicted by the Targetscan. **G**, **H** The binding relationship was identified by a dual-luciferase reporter assay. **I**, **J** Western blot analysis was used to evaluate TCTN1 protein expression in TE-1 and Eca-109 cells after miR-1229-3p overexpression and knockdown. The assay was performed with three independent biological replicates. *P < 0.05

Subsequently, TCTN1 expression was significantly elevated in ESCC tissues and cells (Fig. 5A–C). miR-1229-3p and TCTN1 expression had a negative correlation (Fig. 5D), while TCTN1 and circCHSY1 showed a positive correlation (Fig. 5E). Next, miR-1229-3p was found to target the TCTN1 3’-UTR regions (Fig. 5F). Luciferase reporter activity was repressed in TE-1 and Eca-109 cells co-transfected with miR-1229-3p and WT vector but not with the MUT vector (Fig. 5G and H). In addition, miR-1229-3p decreased TCTN1 expression, and miR-1229-3p downregulation enhanced TCTN1 expression (Fig. 5I and J). In summary, miR-1229-3p was an upstream molecule that targeted TCTN1 and negatively regulated TCTN1 expression.

### Overexpression of TCTN1 alleviated the effects of miR-1229-3p in ESCC cells

**Fig. 6:**
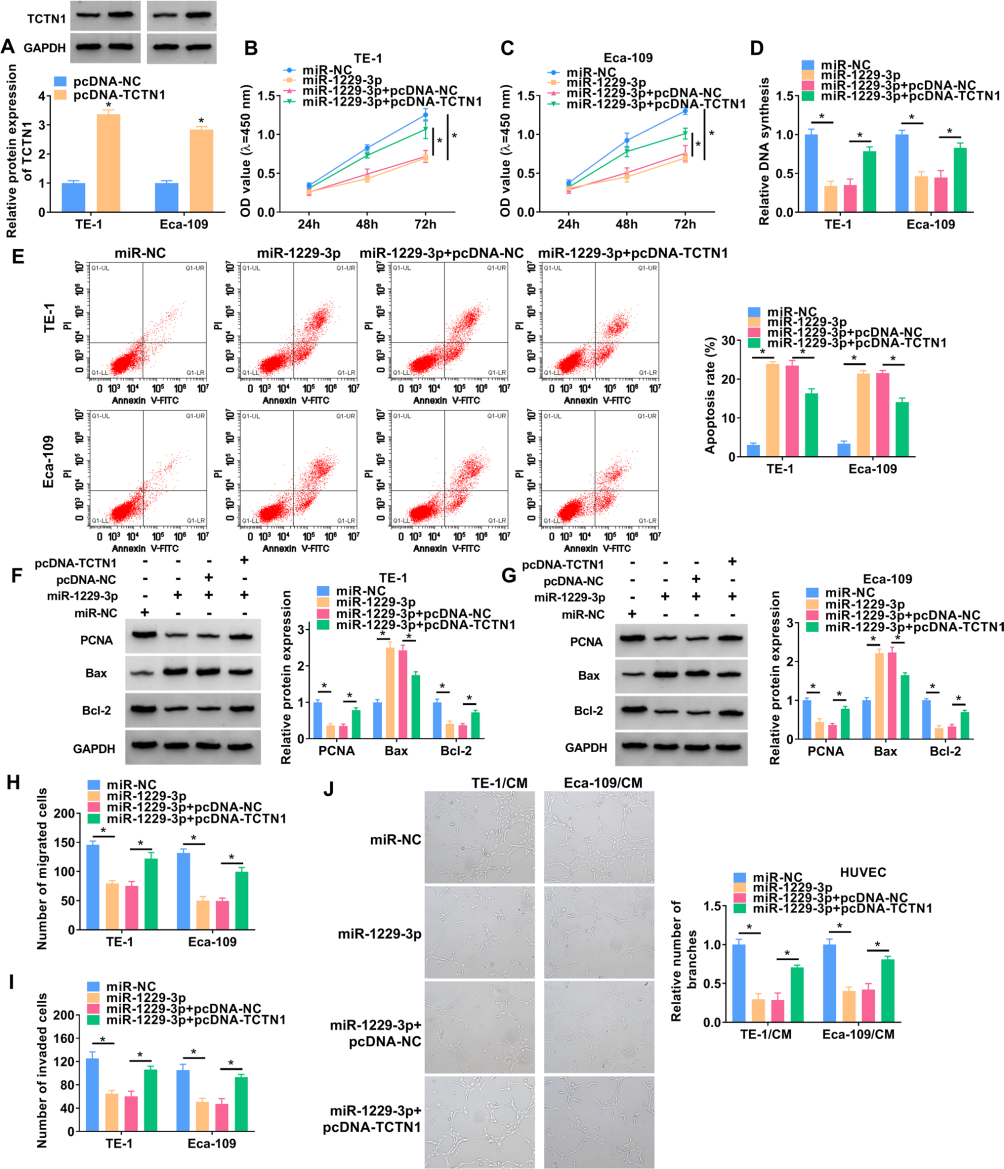
Overexpression of TCTN1 alleviated the effect of miR-1229-3p on proliferation, migration, invasion, and apoptosis in ESCC cells. **A** The protein expressions of TCTN1 were explored using western blot in ESCC cells. **B**–**D** Cell proliferation was assessed by CCK-8 assay **B** and **C** and EdU assay (**D**). **E** Flow cytometry was used to analyze cell apoptosis. **F**, **G** The relative protein expressions of PCNA, Bax and Bcl-2 were determined with western blot. **H**–**J** The ability of cell migration, invasion and angiogenesis was detected by transwell assay and tube formation assay. The assay was performed with three independent biological replicates. *P < 0.05

The function of the miR-1229-3p/TCTN1 axis in ESCC cells was further explored through rescue experiments. The protein level of TCTN1 was significantly increased after transfection with TCTN1 overexpressed plasmid (Fig. 6A). CCK-8, EdU, and flow cytometry experiments showed that TCTN1 overexpression plasmid reversed the effects of miR-1229-3p on cell proliferation and apoptosis (Fig. 6B–G). In addition, miR-1229-3p resulted in significant suppression of migration, invasion, and angiogenesis in both ESCC cell lines, while overexpression of TCTN1 neutralized these effects (Fig. 6H–J).

### CircCHSY1 targeted miR-1229-3p to regulate TCTN1

**Fig. 7:**
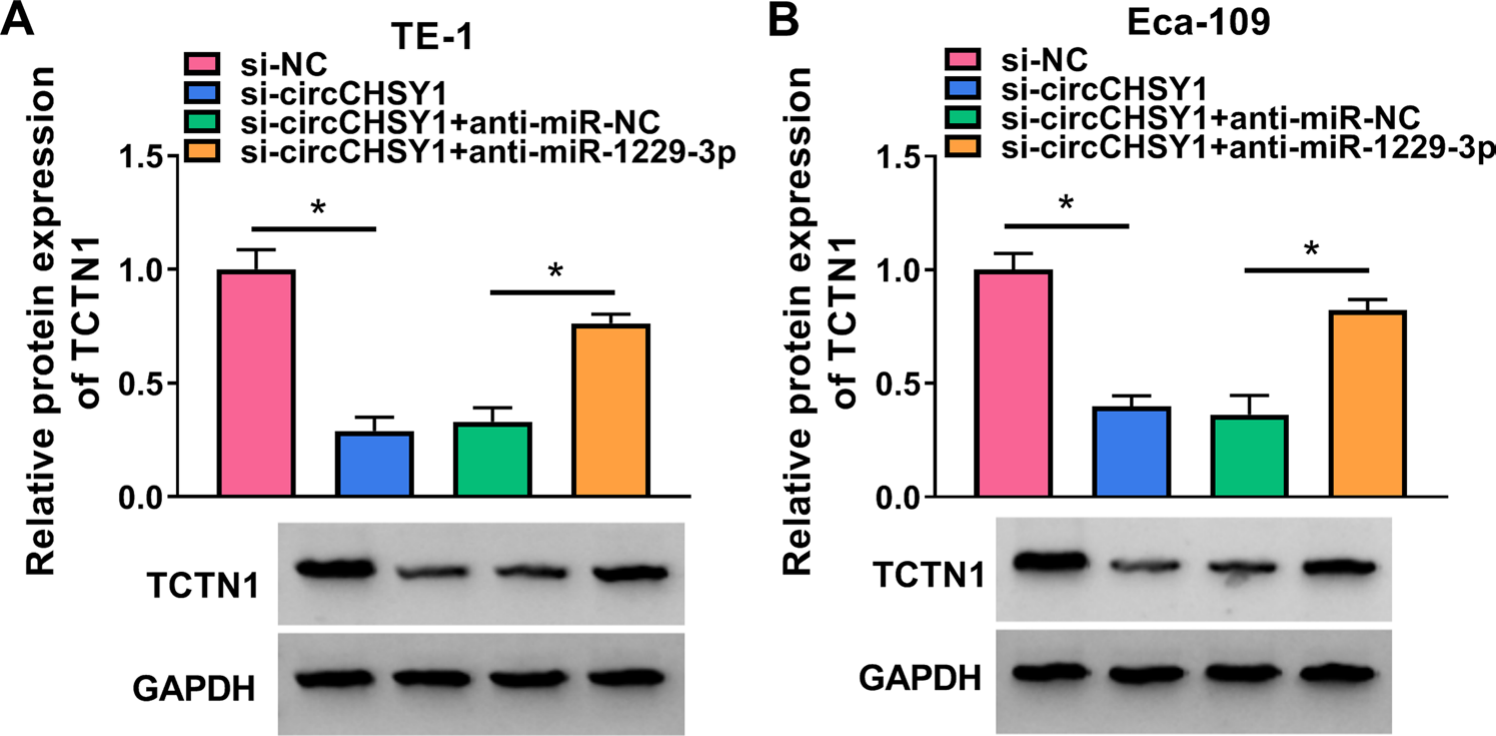
CircCHSY1 targeted miR-1229-3p to promote TCTN1 expression in ESCC cells. (A and B) The protein levels of TCTN1 were examined in TE-1 and Eca-109 cells transfected with si-NC, si-circCHSY1, si-circCHSY1, and anti-miR-NC or anti-miR-1229-3p by western blot. The assay was performed with three independent biological replicates. *P < 0.05

As shown in Fig. 7A and B, the deletion of circCHSY1 led to a decrease in TCTN1 expression, which was reversed by the miR-1229-3p inhibitor.

### Knockdown of circCHSY1 hindered the growth of TE-1 cells in nude mice

**Fig. 8:**
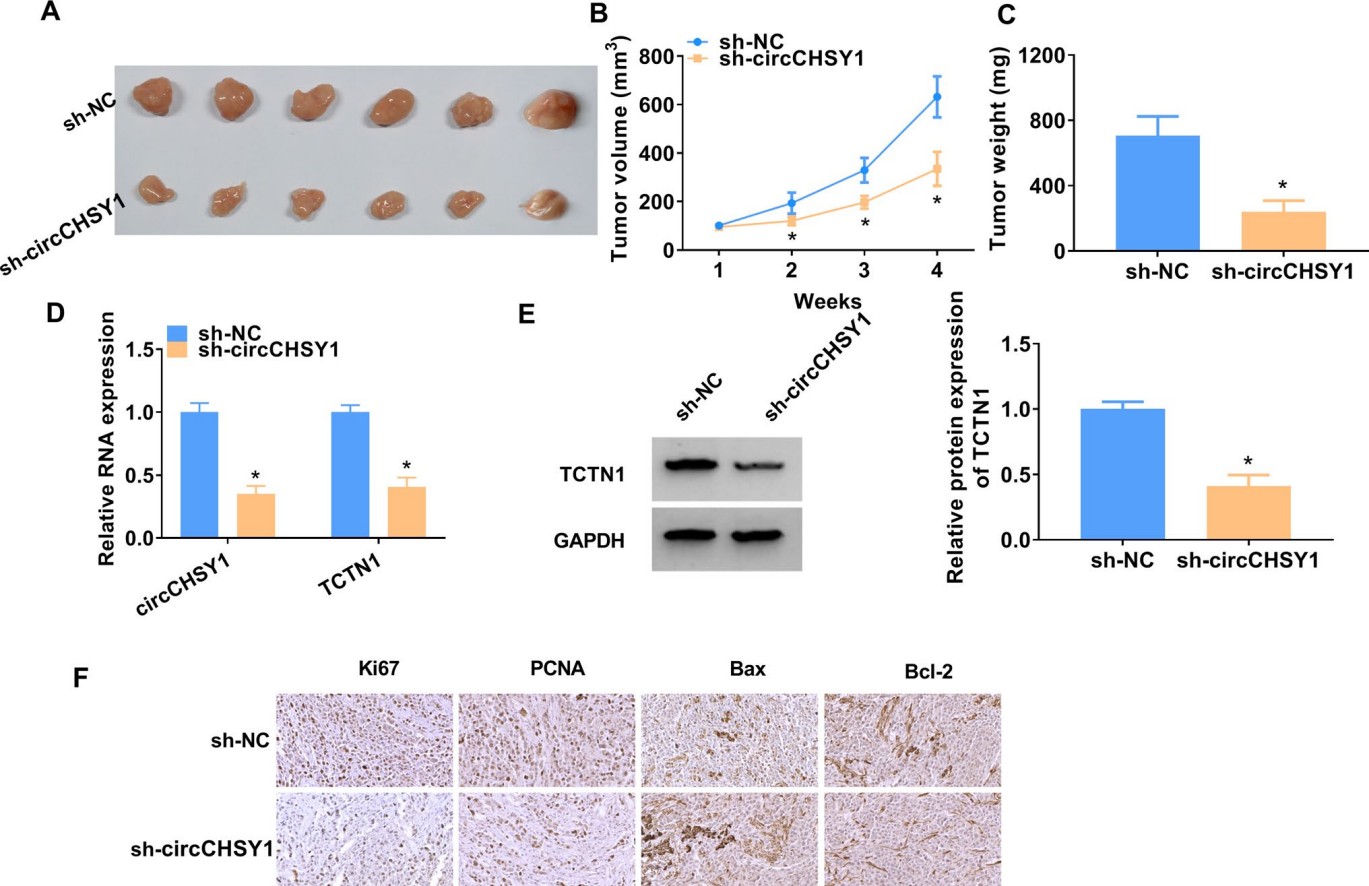
Knockdown of circCHSY1 hindered tumor property of TE-1 cells in nude mice. **A** Representative tumor images were displayed. **B** Tumor volume was measured every 7 days and tumor volume growth curves were plotted. **C** The tumor tissues of the mice were taken out and the tumor weight was calculated. **D**, **E** The expression levels of circCHSY1 and TCTN1 were examined by qRT-PCR and western blot. **F** IHC assay was used to detect the expression of Ki67, PCNA, Bax and Bcl-2 in tumor tissues. The assay was performed with six independent biological replicates. *P < 0.05

Tumor size and tumor weight were suppressed by decreasing circCHSY1 expression (Fig. 8A–C). Besides, circCHSY1 and TCTN1 expression were decreased in the sh-circCHSY1 group (Fig. 8D and E). sh-circCHSY1 treatment decreased Ki-67, PCNA, and Bcl-2 levels and increased Bax level in tumors (Fig. 8F).

## Discussion

CircRNAs can act as oncogenes or suppressor genes in various cancers by modulating signaling pathways [19]. In ESCC, circ-SLC7A5 [20], circ-LRP6 [21] and circ-TTC17 [22] have been reported to play tumor-promoting roles, but circFoxo3 [23] and circSMAD7 [24] act tumor-inhibiting roles. In a previous study, researchers revealed that circCHSY1 was significantly overexpressed in ESCC [13]. Although some evidence points to the involvement of circCHSY1 in disease progression through circ_0005019 [25], further research is required to gain a deeper understanding of the intricate networks regulated by circCHSY1. This might help us to develop clinical diagnostic reagents or seek more therapeutic targets. In this research, we discovered that circCHSY1 expression was increased in ESCC patients and cell lines. We observed that circCHSY1 silencing restrained cell migration, invasion, and angiogenesis, while also promoting apoptosis rate. Also, circCHSY1 silencing inhibited tumor growth in a xenograft mouse model using TE-1 cells.

MiR-1229-3p is involved in the progression of many cancer tumors such as colorectal cancer, glioma, and hepatocellular carcinoma [26–28]. In this study, we discovered this miRNA in tumor tissues and cells was significantly reduced, consistent with previous findings [29]. Moreover, miR-1229-3p showed a significant negative correlation with circCHSY1. CircRNA functions include miRNA sponge, alternative splicing, and regulation of gene transcription [30, 31]. CircRNA indirectly affects gene expression through direct absorption of miRNA [32]. Based on the above theories, we wondered whether circCHSY1 could sponge miR-1229-3p. Interestingly, in this project, miR-1229-3p inhibitor could reverse circCHSY1-mediated changes in ESCC cells’ function to a certain extent.

TCTN1 is a protein composed of 587 amino acids [33]. It belongs to the tectonic family and is closely related to the Hedgehog signaling pathway [34]. Early reports indicated that TCTN1 was associated with numerous tumors including gastric cancer [35], human thyroid cancer [36], as well as ESCC [37]. In this topic, TCTN1 was abnormally elevated in ESCC tissues. TCTN1 was negatively modulated via miR-1229-3p and could participate in the changes in cell function caused by miR-1229-3p. Meanwhile, the absence of circCHSY1 reduced TCTN1 expression, whereas the inhibitor of miR-1229-3p reversed this phenomenon. These findings fully demonstrated that the novel signal axis circCHSY1/miR-1229-3p/TCTN1 was closely related to the regulation of ESCC.

However, there are some limitations to this research. Firstly, all clinical samples were collected from one hospital, and the sample size is relatively small. It is recommended to collect more samples from different hospitals in the future. In addition, the mechanism by which TCTN1 regulates ESCC progression has not been studied, and further experiments using other ESCC cell lines should be performed according to the methods described in this manuscript. The results show that the circCHSY1/miR-1229-3p/TCTN1 axis has a direct or indirect association with Bax, Bcl-2 and PCNA expression, and the relationship may have important influence on cell cycle regulation and apoptosis process. Further research is needed to test these hypotheses and explore their specific mechanisms of action.

## Conclusion

**Fig. 9:**
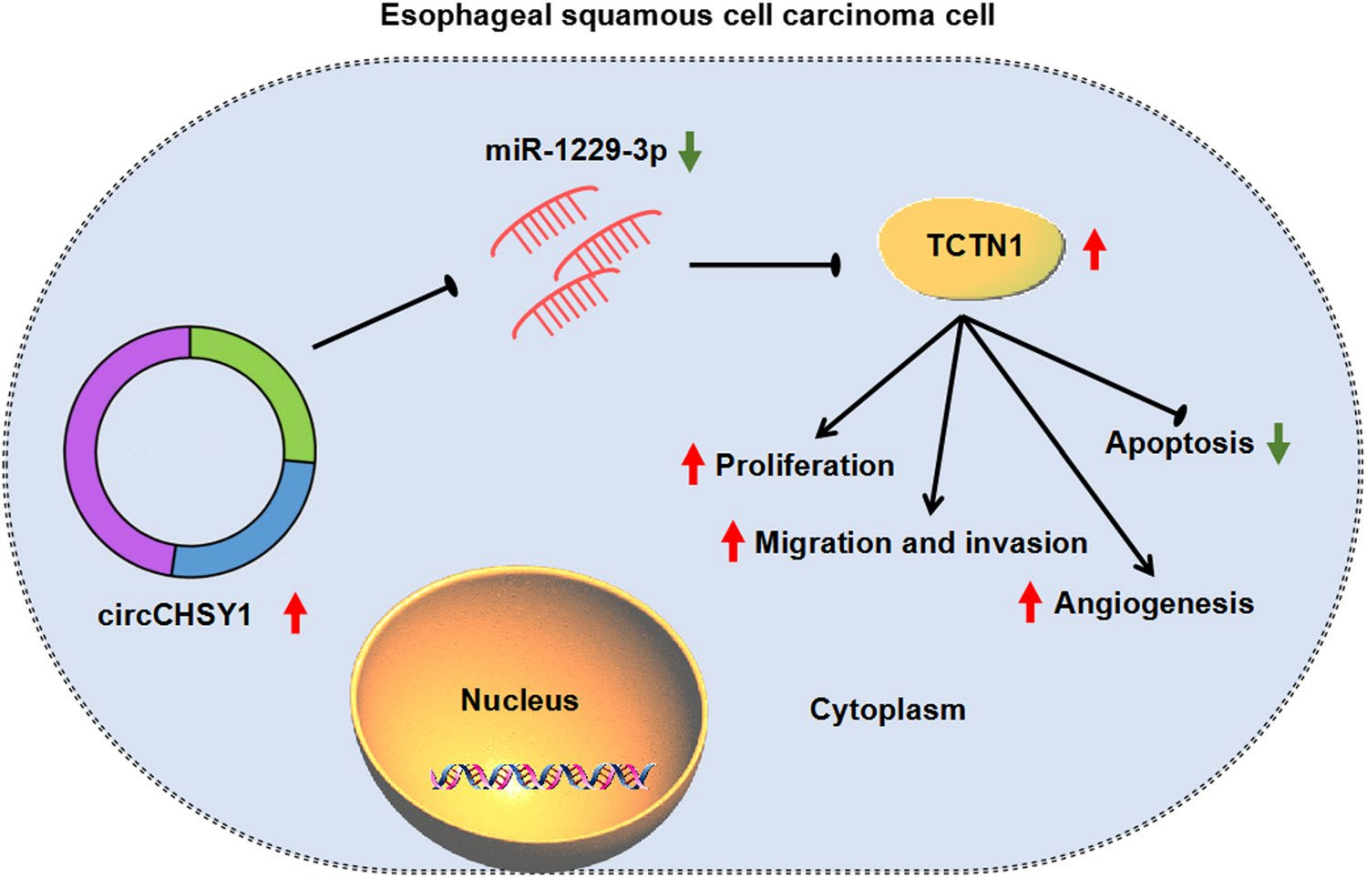
The illustration showed the mechanism of circCHSY1 in regulating ESCC cell tumor properties. ESCC development involved circCHSY1 overexpression, and the increased expression of circCHSY1 induced TCTN1 production through miR-1229-3p, thereby promoting cell proliferation, migration, invasion, and angiogenesis and inhibiting cell apoptosis

In brief, this paper disclosed that circCHSY1, which was abnormally elevated in ESCC, could contribute to the malignant progression of ESCC. This effect was achieved through the regulation of circCHSY1 in the miR-1229-3p/TCTN1 pathway. More precisely, ESCC progression involved the increased circCHSY1 expression in ESCC cells, and circCHSY1 downregulation induced TCTN1 expression by segregating miR-1229-3p (Fig. 9). Accordingly, circCHSY1 could act as a promising therapeutic target in terms of ESCC. These results mean that we have a deeper understanding of how ESCC occurs, which could help develop more effective treatments. The development of new treatment strategies or drugs will provide more treatment options for patients with ESCC and help improve patient survival.

## Data Availability

The datasets used and analyzed during the current study are available from the corresponding author on reasonable request.
